# The effect of low-molecular-weight heparin combined with amikacin on the coagulation function and bacterial clearance in the treatment of patients with severe senile pneumonia

**DOI:** 10.12669/pjms.39.1.6627

**Published:** 2023

**Authors:** Aihong Luo, Ya Liu

**Affiliations:** 1 Aihong Luo, Department of ICU, Shandong Provincial Third Hospital, Cheeloo College of Medicine, Shandong University, Jinan, Shandong Province, P.R. China; 2 Ya Liu, Department of ICU, Shandong Provincial Third Hospital, Cheeloo College of Medicine, Shandong University, Jinan, Shandong Province, P.R. China

**Keywords:** Amikacin, Bacterial clearance, Low molecular weight heparin, Severe pneumonia

## Abstract

**Objective:**

To analyze the effect of the combined low-molecular-weight heparin (LMWH) and amikacin treatment on the bacterial clearance and changes in coagulation function in patients with severe pneumonia (SP).

**Methods:**

A single-center retrospective observational study was conducted. Medical records of 526 elderly patients with SP admitted to the ICU Department of Shandong Provincial Third Hospital from February, 2018 to December, 2021 were reviewed and 342 patients were identified. The patients were divided into two groups according to the treatment records: the study group (175 patients received LMWH combined with amikacin) and the control group (167 patients received amikacin). Changes in coagulation indexes before and after the treatment, as well as bacterial clearance rate and clinical efficacy after the treatment were compared between the two groups.

**Results:**

There was no significant difference in prothrombin time (PT), D-Dimer (D-D), antithrombin III (AT-III) and fibrinogen (FIB) levels between the two groups before the treatment (*P*>0.05). After the treatment, levels of PT, D-D and FIB in the two groups decreased and the level of AT-III increased(*P*<0.05). Levels of PT, D-D and FIB in the study group were lower, and the level of AT-III was higher (*P*<0.05) that n in the control group. Bacterial clearance rate in the study group was (86.19%), higher than that in the control group (72.25%) (*P*<0.05). The total clinical effective rate of the study group (93.14%) was significantly higher than that of the control group (79.04%) (*P*<0.05).

**Conclusions:**

Combining LMWH with amikacin in the treatment of elderly SP patients can improve the coagulation function and bacterial clearance, can promote the recovery of patients and has a good clinical application value.

## INTRODUCTION

Pneumonia is one of the leading infectious causes of death in elderly patients that easily progresses into severe pneumonia (SP) due to comorbidities and waning immunity.^1^ SP is associated with high rates of ventilation dysfunction, respiratory failure and high mortality and requires ICU admission.^2^ Amikacin is a semi-synthetic aminoglycoside antibiotic that is used for treating severe Gram-negative infections. However, systemic administration of amikacin is associated with serious nephrotoxicity, thus limiting its large-dose clinical application.^3^,^4^ Due to the inflammatory reaction to infection, SP is often accompanied by macrovascular and microvascular thrombosis and lung tissue injury.^5^

Low molecular weight heparin (LMWH) is a commonly used anticoagulant in clinic, and is often used to reduce the risk of pulmonary vascular microthrombosis.^6^provide guidance on the use of currently available testing of the coagulation cascade, and help practitioners use anticoagulation and procoagulants appropriately in patients with cirrhosis.\nMETHODS: This review is framed around the best practice points, which were derived from the most impactful publications in the area of coagulation in cirrhosis and agreed to by all authors. BEST PRACTICE ADVICE 1: Global tests of clot formation, such as rotational thromboelastometry, thromboelastography, sonorheometry, and thrombin generation, may eventually have a role in the evaluation of clotting in patients with cirrhosis, but currently lack validated target levels. BEST PRACTICE ADVICE 2: In general, clinicians should not routinely correct thrombocytopenia and coagulopathy before low-risk therapeutic paracentesis, thoracentesis, and routine upper endoscopy for variceal ligation in patients with hepatic synthetic dysfunction-induced coagulation abnormalities. BEST PRACTICE ADVICE 3: Blood products should be used sparingly because they increase portal pressure and carry a risk of transfusion associated circulatory overload, transfusion related acute lung injury, infection transmission, alloimmunization, and/or transfusion reactions. BEST PRACTICE ADVICE 4: The following transfusion thresholds for management of active bleeding or high-risk procedures may optimize clot formation in advanced liver disease: hematocrit ≥25%, platelet count >50,000, and fibrinogen >120 mg/dL. Commonly utilized thresholds for international normalized ratio correction are not supported by evidence. BEST PRACTICE ADVICE 5: Thrombopoietin agonists are a good alternative to platelet transfusion, but require time (about 10 days Studies have showed that LMWH can alleviate renal inflammatory response in patients with sepsis.^7^ In addition, heparin also has an anti adhesion effect, which may help amikacin play a bactericidal role when combined with antibiotics such as amikacin.^8^

At present, there are only few clinical studies on the combined application of LMWH and amikacin. The main goal of this study was to retrospectively collect the clinical data of elderly SP patients treated with amikacin and LMWH and analyze the therapeutic effect of the combined application of the two drugs to provide reference for clinical treatment schemes for elderly SP patients.

## METHODS

In this single-center retrospective observational study, the medical records of 526 elderly patients with SP in the ICU Department of Shandong Provincial Third Hospital from February, 2018 to December, 2021 were reviewed, and 342 patients were finally identified. The patients were divided into two groups: the study group (175 patients received LMWH combined with amikacin) and the control group (167 patients received amikacin).

Definition of SP: There is currently no unified definition of SP as it varies from country to country, but the clinical diagnosis of SP is based on patients’ needs of mechanical ventilation or septic shock; or other clinical symptoms such as respiratory rate > 30 breaths per minute, PaO_2_/FiO_2_ < 250, multilobar infiltrates, conscious disturbance, uremia, leukopenia, thrombocytopenia, hypothermia or hypotension requiring fluid resuscitation.^9^

### Inclusion criteria:

- Patients met the relevant diagnostic criteria based on the guidelines for the management of adult community-acquired pneumonia issued by the American Thoracic Society;^9^
- Age ≥65 years old;
- No liver dysfunction;
- Complete clinical medical records;
- All of them were not resistant to amikacin.

### Exclusion criteria:

- History of immune deficiency diseases;
- Combined with tuberculosis, active bleeding, thromboembolism and other serious diseases;
- Combined with other site infection or malignant tumor;
- Accompanied by organ failure such as heart or kidney failure;

### Ethical Approval:

This study was approved by the medical ethics committee of our hospital (Approval number: LL202108024, Date: 2021-08-15).

All patients were treated with strict respiratory isolation and routine symptomatic support, including adequate airway drainage, mechanical ventilation, sedation and analgesia, anti-infection therapy, correction of water electrolyte disorder and nutritional support. Amikacin (Zhejiang sincere Pharmaceutical Co., Ltd., H200321) was administered by intravenous drip, 7.5 mg/kg, once a day, for ten days. LMWH sodium (Qilu Pharmaceutical Co., Ltd, H20030428), 4000U, was administered as subcutaneous injection, once a day for ten days. The following basic clinical data of patients and relevant indicators were collected before and after the treatment:

Coagulation function indexes before and after the treatment, including prothrombin time (PT), D-Dimer (D-D), antithrombin III (AT-III), fibrinogen (FIB). Detection method: 3ml of peripheral venous blood was collected into a vacuum tube containing 0.09% sodium citrate anticoagulant, centrifuged at 3000r/minute for 10 minutes, and the levels of coagulation function indexes in the supernatant were measured within 60 minutes using relevant kits according to manufacturer’s instructions and full-automatic hemagglutination analyzer (STAGO compact type in France) for detection.

### Bacterial clearance effect:

10 sputum samples after treatment were inoculated and cultured; suspicious bacteria after culture were selected and stained with Gram staining. Pathogenic bacteria were detected by microbial identification and drug sensitivity analysis. In cases of bacteriological culture negative for two consecutive times, the bacterial clearance was considered complete. If bacterial culture after the treatment had the same pathogenic bacteria present, it was considered as not cleared. If one of the two or more kinds of pathogenic bacteria originally cultured were cleared after the treatment, it was considered as partial clearance.

### Cultivation and identification of pathogenic bacteria:

11 The NEW ATB automated microbial identification system (Produced by Meirier, France) is used for identification of pathogenic bacteria. The drug sensitivity reagent is provided by Beijing Weitai Biotechnology Co., Ltd. The quality control strains include Pseudomonas aeruginosa (Brand ATCC, No.: 27853) and Acinetobacter baumannii (Brand ATCC, No.: 19606).

### Clinical efficacy was classified as follows:

12 Remarkable effect was considered if the symptoms and signs of cough and body temperature in elderly patients with SP returned to normal, and the chest X-ray examination showed that the inflammation was completely absorbed; *Effective:* the patient’s cough is relieved, the body temperature is decreased or normal, and the X-ray chest film shows that the inflammation is absorbed; *Ineffective:* the patient’s cough is aggravated, and the body temperature has not decreased or even increased. The results of chest X-ray examination suggest that the inflammation is completely unabsorbed, or even aggravated.

### Statistical Analysis:

SPSS 22.0 was used for analysis of the data; [n (%)] was used to represent non grade count data with *χ^2^* as the test method. T-test and (*χ̄*±*S*) were used to represent the measurement data. *P*<0.05 indicated statistically significant difference.

## RESULTS

A total of 342 elderly patients with SP were included in this study, among whom 175 patients received LMWH combined with amikacin (study group) and 167 patients received amikacin (control group). The mean age of these 342 patients was 73.10±4.64 years (177 males and 165 females). The clinical characteristics of the patients are shown in Table-I. There was no significant difference in the basic clinical data between the two groups (*P*>0.05).

**Table-I:** Comparison of general conditions between the two groups[n(%),*X̅*±*S*].

Group	n	Gender (%)		Age (year)	Course of disease (day)	Basic diseases		
	
		Male	Female			Cardiovascular disease	Cerebrovascular disease	Diabetes
Study group	175	92(52.57)	83(47.43)	72.69±4.72	16.16±3.38	39 (22.29)	49 (28.00)	87 (49.71)
Control group	167	85(50.90)	82(49.10)	73.53±4.52	16.33±3.66	50 (29.94)	40 (23.95)	77 (46.11)
χ^2^/t		0.096		1.672	0.444	2.694		
P		0.757		0.096	0.657	0.260		

The comparison of coagulation indexes between the two groups is shown in Table-II. There was no significant difference in the levels of PT, D-D, AT-III and FIB between the two groups before the treatment (*P*>0.05). After the treatment, the levels of PT, D-D and FIB in the two groups decreased and the level of AT-III increased (*P*<0.05). Levels of PT, D-D and FIB in the study group were lower and the level of AT-III was higher than in the control group (*P*<0.05).

**Table-II:** Comparison of coagulation indexes between the two groups (*X̅*±*s*).

Group	n	PT (s)		D-D (mg/L)		AT-III (%)		FIB (g/L)	

		Before treatment	After treatment	Before treatment	After treatment	Before treatment	After treatment	Before treatment	After treatment
Study group	175	21.42±4.39	12.36±4.19^*^	8.43±2.08	4.10±1.71^*^	66.06±6.88	84.16±5.96^*^	5.51±1.09	3.62±0.98^*^
Control group	167	22.03±4.63	14.47±4.18^*^	8.77±2.18	6.27±1.85^*^	66.73±6.59	78.41±6.19^*^	5.54±1.22	4.63±1.16^*^
t		1.244	4.649	1.480	11.245	0.915	8.757	0.263	8.623
P		0.214	<0.001	0.140	<0.001	0.361	<0.001	0.793	<0.001

The comparison of bacterial clearance between the two groups after treatment is shown in Table-III. The bacterial clearance rate of the study group (86.19%) was significantly higher than that of the control group (72.25%) (*χ^2^*=3.950, *P*<0.05).

**Table-III:** Comparison of bacterial clearance between the two groups [n(%)]

Pathogenic bacteria	Study group				Control group			

	n	Completely clear	Partially cleared	Clearance rate (%)	n	Completely clear	Partially cleared	Clearance rate (%)
Escherichia coli	42	42	0	23.20%	38	25	13	14.45%
Acinetobacter baumannii	36	35	1	19.34%	28	18	10	10.40%
Klebsiella pneumoniae	48	35	13	19.34%	45	31	14	17.92%
Pseudomonas aeruginosa	55	44	11	24.31%	62	51	11	29.48%
Total	181	156	25	86.19%	173	125	48	72.25%

***Note:*** *χ^2^*=10.491, P=0.001.

The comparison of clinical efficacy between the two groups is shown in Table-IV. The total clinical effective rate of the study group (93.14%) was significantly higher than that of the control group (79.04%) (*P*<0.05).

**Table-IV:** Comparison of clinical efficacy between the two groups [n(%)]

Group	n	Remarkable effect	Effective	Ineffective	Total effective rate
Study group	175	98 (56.00)	65 (37.14)	12 (6.87)	163 (93.14)
Control group	167	81 (48.05)	51 (30.54)	35 (20.96)	132 (79.04)
χ^2^					14.334
P					<0.001

## DISCUSSION

This study showed that the coagulation function and bacterial clearance effect of LMWH combined with amikacin in the treatment of severe senile pneumonia (SP) were higher than that of amikacin alone (P<0.05). SP is one of the clinical high incidence diseases, which is mostly caused by Inflammatory reaction caused by pulmonary infection is considered one of the major causes of SP and SP-associated high mortality.^13^ The inflammatory response in patients with SP leads to the injury of pulmonary vascular endothelial cells, the increase of the expression of fibrinolytic inhibitory factors and procoagulant factors, resulting in the activation of the coagulation system, the occurrence of hypercoagulability and local micro thrombosis. The increase in fiber composition of lung tissue, and the occurrence of hypoxemia, hypoxemia and uncontrolled lung inflammation can lead to lung tissue injury, and in severe cases, to life threatening septic shock.^14^–^1^caused by severe acute respiratory syndrome coronavirus 2, keeps spreading globally. Evidence suggests that a subgroup of patients with severe symptomatology might have cytokine storms, which increases mortality. The use of interleukin-6 (IL-6^6^ Study by Salluh et al.^17^the predictive value of d-dimer and of the presence of associated coagulation derangements in severe community-acquired pneumonia (CAP showed that patients with severe SP often have abnormal coagulation function. Iba et al.^18^the most severely ill patients present with coagulopathy, and disseminated intravascular coagulation (DIC also found evidence of coagulation disorder in patients with severe COVID-19. Pavoni et al.^19^ showed that in COVID-19 patients LMWH treatment causes a release of large levels of vascular endothelial plasminogen activator and promotes fibrinolysis. Additionally, it has the antioxidative effect and promotes the elimination of oxygen free radical, thus protecting vascular endothelial cells, alleviating blood hypercoagulability and improving the overall coagulation function.^19^

The clinical treatment of elderly SP patients generally includes comprehensive treatment, such as asthma and spasmolytic medication, ventilator support, antibiotics and nutritional support.^20^ Amikacin is an aminoglycoside antibiotic, an acylated derivative of kanamycin A. It has high stability for aminoglycoside inactivating enzyme and works by inhibiting bacterial protein synthesis. The studies by Chen et al.^21^ and Li Bassi et al.^22^ also confirmed that amikacin is relatively stable against aminoglycoside inactivating enzymes produced by intestinal Gram-negative bacteria, will not lose antibacterial activity due to enzyme passivation, and has strong antibacterial effect. Amikacin has a strong killing effect on Gram-negative bacteria, while heparin can provide a convenient basis for Amikacin to exert its bactericidal effect by inhibiting the adhesion of pathogens.^8^,^22^ The combined application of heparin and amikacin may, therefore, play a synergistic role. In support of this hypothesis, in our study, the bacterial clearance rate of the study group (86.46%) was significantly higher than that of the control group (74.29%), indicating that LMWH combined with amikacin can achieve higher bacterial clearance rate.

### Limitations:

It is a single center study, and the long-term efficacy in elderly SP patients has not been observed. Further multi-center studies with extended follow-up clinical efficacy are needed to further confirm the conclusion of this report.

## CONCLUSION

The application of LMWH combined with amikacin in the treatment of elderly SP patients can significantly improve their coagulation function, improve the bacterial clearance rate and promote the recovery of patients. It has a good clinical application value.

### Authors’ contributions:

**AL & ILL:** Conceived and designed the study, collected the data and performed the analysis, Prepared the manuscript and are responsible for the integrity of the study.
